# Dendritic cells in liver transplantation immune response

**DOI:** 10.3389/fcell.2023.1277743

**Published:** 2023-10-13

**Authors:** Xiaodong Du, Mingqian Li, Chen Huan, Guoyue Lv

**Affiliations:** ^1^ Department of Hepatobiliary and Pancreatic Surgery, General Surgery Center, The First Hospital of Jilin University, Changchun, China; ^2^ Center of Infectious Diseases and Pathogen Biology, Institute of Virology and AIDS Research, Key Laboratory of Organ Regeneration and Transplantation of The Ministry of Education, The First Hospital of Jilin University, Changchun, China

**Keywords:** liver transplantation, dendritic cells, immune rejection, immune tolerance, DC based treatment

## Abstract

Dendritic cells (DCs) are the most powerful antigen presenting cells (APCs), they are considered one of the key regulatory factors in the liver immune system. There is currently much interest in modulating DC function to improve transplant immune response. In liver transplantation, DCs participate in both the promotion and inhibition of the alloreponse by adopting different phenotypes and function. Thus, in this review, we discussed the origin, maturation, migration and pathological effects of several DC subsets, including the conventional DC (cDC), plasmacytoid DC (pDC) and monocyte-derived DC (Mo-DC) in liver transplantation, and we summarized the roles of these DC subsets in liver transplant rejection and tolerance. In addition, we also outlined the latest progress in DC-based related treatment regimens. Overall, our discussion provides a beneficial resource for better understanding the biology of DCs and their manipulation to improve the immune adaptability of patients in transplant status.

## 1 Introduction

In the early 1970 s, Ralph Steinman and others discovered a morphologically and functionally distinct, previously uncharacterized population of cells in their experiments. They called the cells “dendritic cell” (DC) due to their “stellate” or “dendritic” morphology (Steinman and Cohn, 1973). In the liver, DCs are preferentially found in the periportal and pericentral space (Rahman and Aloman, 2013; Eckert et al., 2015). They account for about 1% of non-parenchymal cells and are related to innate and adaptive immunity by promoting and regulating T cell immunity, which are considered to be one of the key regulators in liver immune system (Hsu et al., 2007; Thomson et al., 2020; Bošnjak et al., 2022). DCs continuously flow into the liver from the blood, and circulating DCs are recruited to the hepatic sinusoids by hepatic Kupffer cells in a c-type lectin-dependent manner (Kudo et al., 1997; Uwatoku et al., 2001). Liver normally contains more interstitial dendritic cells than other parenchymal organs (Steptoe et al., 2000), which may be the result of the pathogen associated molecular pattern (PAMP) in portal blood.

Liver transplantation is the most effective treatment for various end-stage liver diseases. The liver is an immune preferential organ (Demetris et al., 2016). In addition to patients undergoing transplantation for autoimmune liver diseases (such as primary cholangitis, primary sclerosing cholangitis, and autoimmune hepatitis), clinically more than 30% of patients can get rid of the use of immunosuppressant (IS) and achieve a state of immune tolerance (Clavien et al., 2017). In liver transplantation, DCs participate in both the promotion and inhibition of the alloreponse by adopting different phenotypes and function. Here, we discussed the origin, maturation, migration and pathological effects of several DC subsets, including the conventional DC (cDC), plasmacytoid DC (pDC) and monocyte-derived DC (Mo-DC), and we summarized the roles of these DC subsets in liver transplant rejection and tolerance. In addition, we also outlined the latest progress in DC-based related treatment regimens.

## 2 The characteristics of DC

### 2.1 The subgroups of DC

According to the current novel, ontogenetic, and functionally relevant simplified classification system, DCs are classified into cDC, pDC, Mo-DC, and Langerhans cell (LC) (Satpathy et al., 2012). cDC can be further divided into two subsets: conventional DC type 1 (cDC1) and conventional DC type 2 (cDC2). cDC can maintain immune homeostasis, simultaneously rapidly respond to local damage, and initiate and guide innate and adaptive immunity (Cabeza-Cabrerizo et al., 2021). pDC act as both innate antiviral effectors and inducers and regulators of adaptive immunity (Fonteneau et al., 2003), regulating the induction and/or maintenance of tolerance to hematopoietic stem cells (HSCs) or allogeneic organ grafts (Rogers et al., 2013). Mo-DC is considered to be the main inflammatory cell type during infection (Geissmann et al., 2003; Serbina et al., 2003). Monocytes differentiate into Mo-DC during inflammation or infection, which cooperate with cDC to induce T cell-mediated immune responses (Bošnjak et al., 2022). Traditionally, LC has been considered a DC population in the epidermis (Schuler and Steinman, 1985; Romani et al., 1989; Wilson and Villadangos, 2004). Therefore, this article will focus on cDC, pDC, and Mo-DC.

### 2.2 DC development

**FIGURE 1:**
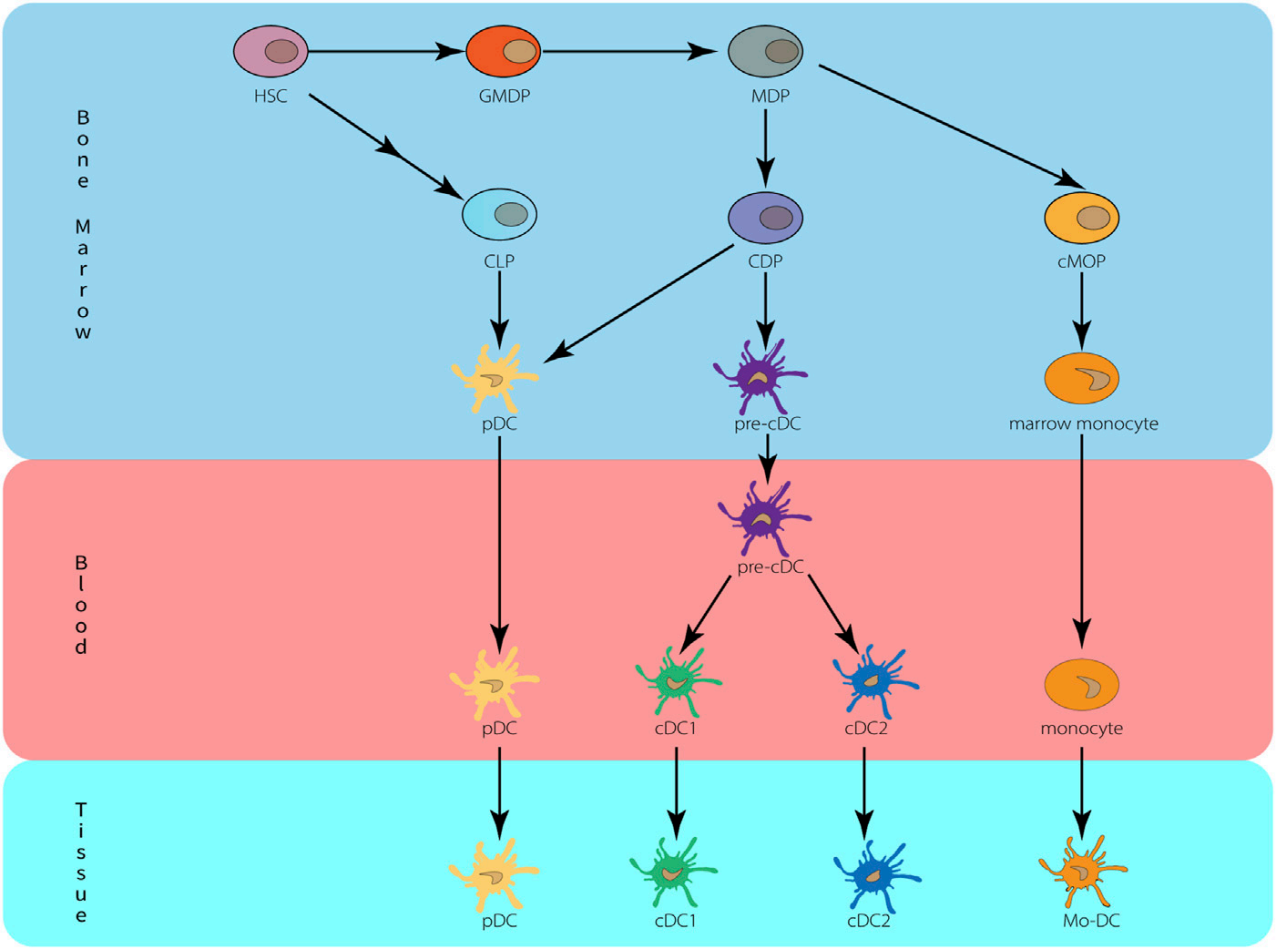
Origin and development of dendritic cells. HSC, hematopoietic stem cells; MDP, monocyte-macrophage DC progenitor; CDP, common dendritic cell progenitor; CLP, common lymphoid progenitor; cMoP, common monocyte progenitors; Mo-DC, monocyte-derived DC.

Human and mouse DC develop from progenitor cells in the bone marrow and then differentiate into distinct subpopulations that are spread across multiple tissues (Durai and Murphy, 2016; Guilliams et al., 2016). HSCs in bone marrow can give rise to granulocytes, monocytes, and DC progenitor cell (GMDP), which in turn give rise to monocyte and macrophage DC progenitor cell (MDP). They give rise to a common DC progenitor (CDP) and a common monocyte progenitor (cMoP) (Fogg et al., 2006; Hettinger et al., 2013). CDP produces classical/conventional DC precursor cell (pre-cDC) and pDC, both cDC1 and cDC2 are derived from pre-cDC and can be found in blood and bone marrow. cMoP produces monocytes in the bone marrow, then enters the peripheral blood, enters the tissue during inflammation or infection and further differentiates into Mo-DC (Hettinger et al., 2013; Que et al., 2020). The development of DC is depicted in Figure 1.

### 2.3 Markers for DC identification

DC subsets differ in the expression of surface markers and transcription factors (Table 1). Mouse cDC could be divided into two subsets: cDC1 was identified as lineage (Lin)^−^ major histocompatibility complex Class II (MHC-II)^+^cluster of differentiation (CD)11c^+^CD103^+^CD11b^−^. cDC2 was identified as Lin^−^MHC-II^+^CD11c^+^CD103^−^CD11b^+^. Human cDC can be subdivided based on the expression of CD1c^+^ [also known as blood dendritic cell antigen (BDCA)1] and CD141^+^ (BDCA3) (Haniffa et al., 2012; Guilliams et al., 2014; Pulendran, 2015). CD141^+^ cDC differentiate and mature under the influence of INF regulatory factor 8 (IRF8), express XC-chemokine receptor 1 (XCR1)^+^, C-type lectin domain containing 9A (CLEC9A)^+^ and B- and T-lymphocyte attenuator 4 (BTLA4)^+^, secrete interleukin 12 (IL-12) to promote T helper 1 cell (T_h_1) differentiation, and also cross-present intracellular antigens (Murphy et al., 2016), these functions are similar to those of cDC1 in mice. CD1c^+^ cDC differentiate and mature under the influence of IRF4, express high levels of CD1b^+^, CD14^+^, and signal-regulatory protein alpha (SIRPα)^+^, and may further differentiate through Krüppel-like factor 4 (KLF4) to promote T helper 2 cell (T_h_2) differentiation or Notch signaling pathway to promote IL-23, thereby contributing to T helper 17 cell (T_h_17) differentiation. These functions are similar to those of cDC2 in mice.

**TABLE 1:** Human and mouse DC subsets with markers and transcription factors.

DC subset	Development, growth and transcription factors	Surface markers mouse	Surface markers human	References
Conventional DC1	FLT3L, GM-CSF, IRF8, ID2, NFIL3, BATF3	Lin^−^MHC-II^+^ CD11c^+^ CD103^+^ CD11b^−^ XCR1^+^ CLEC9A^+^	Lin^−^ HLA-DR^+^ BDCA3/CD141^+^ XCR1^+^ CLEC9A^+^ BTLA4^+^	Bachem et al. (2010), Crozat et al. (2010), Edelson et al. (2010), Poulin et al. (2012), Merad et al. (2013), Guilliams et al. (2016), Anderson et al. (2018)
Conventional DC2	FLT3L, GM- CSF, IRF4, Notch2	Lin^−^ MHC-II^+^ CD11b^+^ CD11c^+^ CD103^-^ SIRPα^+^	Lin^−^ HLA-DR^+^ BDCA1/CD1c^+^ SIRPα^+^ XCR1^-^ CLEC9A^-^	Lewis et al. (2011), Merad et al. (2013), Satpathy et al. (2013), Yin et al. (2021)
Plasmacytoid DC	FLT3L, M-CSF, TCF4(E2-2), IRF8, BCL11A, ZEB2, Spi-B	MHC-II^int^ CD11c^int^ B220^+^ Ly6C^+^ BST2^+^ SiglecH^+^	HLA-DR^+^ CD11c^−^ CD4^+^ BDCA2/CD303^+^ BDCA4CD304^+^ CD123^+^	Colonna et al., 2004; Liu (2005), Cisse et al. (2008), Grajkowska et al. (2017), Reizis (2019)
Monocyte-derived DC	GM-CSF or IL-4 PU.1, IRF4, AHR, NR4A3, NCOR2	MHC-II^+^ CD11C^+^ CD11b^+^ CD64^int^ Ly6C^int^ CCR2^+^ CD209^+^	HLA-DR^+^ CD11c^+^ CD14^int^ CD206^+^ CD1c^+^	Lehtonen et al. (2005), Segura et al. (2013), Schlitzer et al. (2015), Briseño et al. (2016), Menezes et al. (2016), Goudot et al. (2017), Sander et al. (2017), Boulet et al. (2019), Tang-Huau and Segura (2019)

The phenotype of mouse pDC was identified as MHC-II^int^CD11c^int^B220^+^lymphocyte antigen 6 (Ly6)C^+^ bone marrow stromal antigen (BST)2^+^Sialic acid-binding immunoglobulin-type lectin (Siglec)-H^+^ (Colonna et al., 2004). The phenotype of human pDC was identified as human leukocyte antigen–DR isotype (HLA-DR)^+^CD11c^−^CD4^+^ BDCA2^+^BDCA4^+^CD123^+^ (Liu, 2005). Like pDC in mice, human pDC also can secrete type I interferon (IFN-I) and initiate antiviral immunity (Haniffa et al., 2012). The development of pDC depends on FMS-like tyrosine kinase 3 (FLT3) and its ligand FMS-like tyrosine kinase 3 ligand (FLT3L) (Schmid et al., 2010), and macrophage colony-stimulating factor (M-CSF) may also support the generation of pDC (Fancke et al., 2008). Transcription factor 4 (TCF4)(E2-2), IRF8, B-cell lymphoma/leukemia 11A (BCL11A), Zinc finger E-box-binding homeobox 2 (ZEB2), and Spi-B play important roles in the development and maintenance of the pDC phenotype (Reizis, 2019).

The phenotype of mouse Mo-DC was identified as MHC-II^+^CD11c^+^CD11b^+^CD64^int^Ly6C^int^ C-C chemokine receptor 2 (CCR2)^+^CD209^+^ (Schlitzer et al., 2015; Menezes et al., 2016). The phenotype of human Mo-DC was identified as HLA-DR^+^CD11c^+^CD14^int^CD206^+^CD1c^+^ (Segura et al., 2013; Tang-Huau and Segura, 2019). Monocyte differentiation into Mo-DC depends on some key regulators, such as PU.1, IRF4, aryl hydrocarbon receptor (AHR), nuclear receptor 4A3 (NR4A3), and nuclear receptor co-repressor 2 (NCOR2) (Lehtonen et al., 2005; Briseño et al., 2016; Menezes et al., 2016; Goudot et al., 2017; Sander et al., 2017; Boulet et al., 2019).

### 2.4 DC migration and activating T cells

#### 2.4.1 DC migration

A study (Lämmermann et al., 2008) used live cell imaging devices to directly observe the movement of DC sensing and entering afferent lymphatic vessels through wide-field microscopy in a mouse ear skin explant model. DC migration mainly depends on two chemokines: C-C motif ligand 19 (CCL19) and CCL21 (Lämmermann et al., 2008). The chemokine CCL21 is constitutively expressed by lymphatic endothelial cells (LECs) (Saeki et al., 1999; Russo et al., 2016), while its corresponding chemokine receptor CCR7 is induced in mature DC (Sallusto et al., 1998). They are expressed in LECs and lymph node T cell regions and bind to CCR7, which is upregulated on DC after activation (Förster et al., 1999). After approaching the lymphatic vessel, DCs dock with the lymphatic endothelium and enter the lumen. The docking and transport process is mediated by the interaction between lymphatic vessel endothelial hyaluronan receptor 1 (LYVE-1) on the LECs and the hyaluronic acid coating on the surface of the DC bound and organized by CD44 (Johnson et al., 2017; Johnson et al., 2021). Some researchers have used an intravital microscope (IVM) to prove that Rho-associated protein kinase (ROCK) is involved in the process of DC crawling and overall migration in lymph (Nitschké et al., 2012). After entering the initial lymphatic vessels, DCs use lamellipodia to crawl along the lymphatic endothelium and enter downstream by sensing lymphatic flow, and once DCs reach the collecting vessels, they begin to drift freely in lymphocytes (Tal et al., 2011). *In vivo* studies in mice, two-photon microscopy showed that after mature DC entered the lymph node parenchyma, there was a strong directional migration of DC to the T cell area, which was dependent on the expression of CCR7 (Braun et al., 2011), and possibly depend on the presence of CCL21 (Schumann et al., 2010). LECs, derived from the top of the subcapsular sinus (SCS) of lymph nodes, express atypical chemokine receptor 4 (ACKR4), which is a clearance receptor for CCR7 ligands and a few other chemokines. By scavenging CCR7 ligands in the SCS lumen, CCL21 gradients are actively created and drive DC exit from SCS into the lymph node parenchyma (Ulvmar et al., 2014). Imaging of these compartments revealed that ACKR4-expressing LECs create a chemokine gradient through local clearance of CCL19 and CCL21, which triggers directional DC migration (Ulvmar et al., 2014). Within lymph nodes, DCs process and present exogenous peptides in MHC I and II, and these MHC-peptide complexes are recognized by CD8^+^ T cells and CD4^+^ T cells through T cell receptors, respectively (Corse et al., 2011).

DCs migrate to the liver from the lymphatic or blood circulation. After entering the liver, Kupffer cells selectively capture DCs and transport them to the draining hepatic lymph nodes through the liver sinus-lymph pathway (Matsuno et al., 1996; Kudo et al., 1997; Matsuno and Ezaki, 2000). Blood-derived DCs are attracted by CCL3 on Kupffer cells and extravasate from the endothelial pores of liver sinus into the Disse space (Sato et al., 1998). The DCs then migrate to the portal vein area through the CCR7-CCL21 system and interact with T cells to create portal tract-associated lymphoid tissue (PALT) (Yoneyama et al., 2001). IL-1 receptor antagonist (IL-1RA), derived from hepatocytes, directly activates the IL-1 receptor (IL-1R)/toll-like receptor (TLR) signaling pathway of activated DCs to regulate this migration by inducing the expression of CCR7 (Iizasa et al., 2005).

#### 2.4.2 Activating T cells

DC carrying antigen in lymph node T cell area can be effectively scanned by naive T cells. When naive T cells encounter cognate antigens in MHC-peptide complexes on the surface of DCs, naive T cells are activated and begin to proliferate rapidly (Bošnjak et al., 2022). The priming of T cells generated in lymph nodes occurs in three distinct stages, which can be distinguished by contact time and migration speed (Mempel et al., 2004; Miller et al., 2004). The first stage occurs approximately 8 h after T cells enter the lymph nodes, and the contact time between T cells and DCs are very short, usually no more than 30 min, and this stage is characterized by short contact between T cells and many DCs, mainly around high endothelial blood vessels. Chemokine CCL21 and intercellular adhesion molecule 1 (ICAM-1) played an important role in their brief contact (Bromley and Dustin, 2002; Friedman et al., 2006). The second stage occurs between approximately 8 h and 24 h, and the binding formed by T cells and DCs is stable and lasts for more than 1 h, and this stage is characterized by the formation of mature synaptoid contact areas between T cells and DCs. Adhesion molecules such as CD2 (Binder et al., 2020), lymphocyte function associated 1 (LFA-1) and ICAM-1 (Bromley and Dustin, 2002), as well as cytokines (Mempel et al., 2004) such as CD40L, IL-2 and interferon gamma (IFN-γ) play a role in the formation of mature synaptoid contact areas. The third stage occurs 24 h after the T cells enter the lymph nodes, the T cells separate from the DC, and the T cells rapidly migrate and proliferate, while continuing to make short contact with the DC. Cytokines (Mempel et al., 2004) such as CD40L, IL-2 and IFN-γ play a role in their short contact. The scavenger molecule Clever-1 (also known as Stabilin-1 and FEEL-1) expressed by LECs was shown to affect the migration of DC from the skin to the draining lymph node (dLN), the maturation state of the migrating DC and its ability to induce T-cell proliferation (Tadayon et al., 2021). Moreover, DC contributes to creating a cytokine environment (such as IL-10, IL-12, IL-17, IL-22, IL-23, IFN-I, tumor necrosis factor [TNF]) that drives naive T cells to differentiate into different types of effector T cells (Satpathy et al., 2012).

### 2.5 DC subpopulations in liver homeostasis

In the liver under homeostasis, hepatic DCs account for approximately 1% of the non-parenchymal hepatic cells and are a diversified population of hepatic APCs linked to innate and adaptive immunity and considered as key modulator of hepatic immune system (Hsu et al., 2007). The conventional hepatic DCs located at the periportal region and central veins, whereas the plasmacytoid hepatic DCs are located within the liver parenchyma (Jomantaite et al., 2004). The cDCs primarily serve as APCs, aiding in the activation and regulation of hepatic immune responses. The pDCs can generate a large amount of IFN-I [IFN-alpha (IFN-α) and IFN-beta (IFN-β)], which are crucial in inhibiting viral replication and promoting antiviral immune responses (Villadangos and Young, 2008; Haniffa et al., 2012). Additionally, pDCs can modulate the functions of other immune cells by producing cytokines and chemical mediators, such as activating NK cells and T cells (Brewitz et al., 2017). What’s more, Mo-DCs play important roles in immune regulation, anti-inflammatory effects, and immune surveillance in the liver homeostasis (León et al., 2007; Mildner et al., 2013; Plantinga et al., 2013). They also contribute to maintaining immune balance and protecting the liver from infection and damage (Boyette et al., 2017; Wolf et al., 2019).

## 3 cDC in the liver transplantation immune response

### 3.1 cDC in the liver transplant rejection

Acute cellular rejection (ACR) occurs in up to 30% of patients within the first year after liver transplantation, and the occurrence of ACR is associated with an increased risk of graft failure, graft failure-related death, and all-cause death (Levitsky et al., 2017). ACR is mainly a cellular immune response mediated by T cells after liver transplantation. DCs, as the most important APCs in priming T cells, they present allogeneic antigens to the cognate T-cell receptor (TCR) to activate them, thus initiating cellular immune responses (Yu et al., 2012; Ueta et al., 2021). Under homeostatic conditions, hepatic DCs exhibit an immature/anti-inflammatory phenotype, T cells are barely activated, and are less immunogenic (Bamboat et al., 2009). However, after liver transplantation, DC gradually matured under the stimulation of inflammatory factors such as TNF alpha (TNF-α), IFN-β, IFN-γ, IL-6, and prostaglandin E_2_ (PGE2). The mature DCs initiate immune responses by inducing T cell proliferation and activation (Reis e Sousa, 2006). Mature DCs are characterized by high expression of MHC-II molecules, costimulatory molecules (such as CD80, CD86 and CD40), chemokine receptors (such as CCR7), adhesion molecules (such as CD62L), and enhanced producing proinflammatory cytokines (such as TNF- α, IL-12) (Penna et al., 2002; Steinman and Banchereau, 2007; Colvin et al., 2008). The production of proinflammatory cytokines due to ischemia-reperfusion injury (IRI) during transplantation is considered to be a key factor involved in the maturation of donor DCs and their migration to recipient secondary lymphoid tissues (Lechler et al., 2001). After transplantation, danger signals mediated by pattern recognition receptors (PRRs) immediately activate DCs, leading to maturation, upregulation of costimulatory molecules, secretion of proinflammatory cytokines, and cytotoxicity (LaRosa et al., 2007).

After liver transplantation, the MHC-peptide complex on donor DC is presented to the host T cells, inducing T cell activation to initiate the immune response, which is a key step leading a donor-direct alloresponse (Hughes et al., 2020; Ronca et al., 2020). It is currently believed that there are three ways of allorecognition (Duneton et al., 2022): the direct pathway is that the donor-derived DC presents the donor MHC-antigen complex to the recipient T cells, the indirect pathway is that the recipient DCs capture donor antigen and present it to the recipient T cells, the semi-direct pathway refers to the presentation of the intact donor MHC I-antigen complex to recipient CD8+T cells in secondary lymphoid organs by “cross-modifying” receptor-derived DCs (it means that recipient DCs have acquired MHC-antigen complexes).

Extracellular vesicles (EVs) with the characteristics of exosomes plays an important role in the semi-direct pathway (Liu et al., 2016; Marino et al., 2016). Exosomes are 70–120 nm EV originated in the endocytic compartment of living cells, which have been shown to transfer proteins and RNAs between cells (Colombo et al., 2014; Robbins and Morelli, 2014). The previous view was that acute graft rejection was related to the migration of immunogenic passenger DCs to recipient lymphoid tissues (Lechler et al., 2001), now the exosomes, as a new mode of intercellular communication, have been shown to participate in IRI and immune regulation early after organ transplantation (Liu et al., 2016; Zheng et al., 2018; Hughes et al., 2020).

### 3.2 cDC in the liver transplant tolerance

In addition to initiating immune responses, cDC also plays a role in inducing and preserving immune tolerance. Lutz *et al.* suggested that cDC that induces tolerance is in a semi-mature state, while the induction of T cell responses requires complete DC maturation (Lutz and Schuler, 2002). DCs have been shown to be essential for maintaining central and peripheral tolerance through immune bias, inducing T cell weakness, promoting T cell apoptosis, and inducing regulatory T cells (Tregs) (Baroja-Mazo et al., 2016). Allogeneic organ transplantation can produce high frequency of allogeneic T cells, and the loss of donor reactive T cells is the key to induce transplantation tolerance. DCs can eliminate donor reactive T cells by inhibiting signaling or producing apoptotic factors (Lu et al., 1994; Süss and Shortman, 1996; Lu et al., 1999).

In the liver microenvironment, the promotion of tolerance by cDCs may be associated with hepatic stellate cells, which activate multiple immunosuppressive circuits in cDCs by secreting cytokines and chemokines (such as IL-6, CCL2, and CCL3) and all trans retinoic acid (ATRA) (Bhatt et al., 2014). These cytokines and chemokines activate the signal transducer and activator of transcription 3 (STAT3) signaling pathway in cDCs and promote apoptosis elimination and tolerance induction of effector T cells by upregulating indoleamine 2,3-dioxygenase (IDO) in a programmed death-ligand 1 (PD-L1)-independent manner (Sumpter et al., 2012). IDO inhibits effector T cells and supports Treg cells by decomposing tryptophan, leading to local tryptophan deficiency and secretion of kynurenine (Mellor et al., 2002; Favre et al., 2010; Higashitani et al., 2013). In addition, ATRA can induce arginase 1 (ARG1) and inducible nitric oxide synthase (iNOS) in cDCs, and both arginine catabolism and nitric oxide (NO) secretion can inhibit T cells (Bhatt et al., 2014).

Under homeostatic conditions, mouse and human hepatic DCs are tolerant (Rastellini et al., 1995; Thomson and Lu, 1999; Bamboat et al., 2009). Compared with DCs from extrahepatic tissues, hepatic DCs exhibit an immature phenotype with low expression of MHC-II and costimulatory molecules (Lu et al., 1994), and they exhibit lower endocytic capacity and are poor stimulators of T cells (Lukacs-Kornek and Schuppan, 2013). In addition, hepatic DCs also exert tolerance through the production of anti-inflammatory PGE2, which can upregulate IDO, enhance the secretion of IL-10 and induce the generation of Tregs (Raïch-Regué et al., 2014). Graft infiltrated host DCs expressed high levels of PD-L1, leading to depletion of CD8^+^ T cells and induction of immune tolerance, suggesting that hepatic DCs have a tolerogenic function after liver transplantation, which was confirmed in a liver transplantation model (Ono et al., 2018). Hepatic cDCs are also less mature in phenotype and function than secondary lymphoid tissues DC. *In vivo* experiments in mice, cDCs from the liver showed reduced ability to activate allogeneic naive T cells (Pillarisetty et al., 2004; De Creus et al., 2005; Abe et al., 2006). In addition, hepatic cDCs can also induce immune tolerance by affecting the function of T cells. In mouse experiments, hepatic cDCs were injected into allogeneic recipients and found to induce IL-10 secretion of T cells (Khanna et al., 2000). Interestingly, exosomes can also exhibit immunosuppressive effects to affect the immunogenicity of allografts in liver transplantation (Mastoridis et al., 2021). In this study, the cross-dressed sEVs (exosomes) showed the ability to inhibit the proliferation of CD8^+^ T cells in liver transplantation. In addition, DCs cocultured with exosomes from liver transplant patients can induce lower costimulatory molecule (CD40) expression, and produce less IL-6 and more IL-10, so they have higher ability to induce DC inhibitory phenotype. In a mouse liver transplantation model (Ono et al., 2018), graft infiltrating DCs cross-dressed by donor exosomes expressed high levels of PD-L1 and significantly inhibited the proliferation of donor reactive CD8^+^ T cells by inducing an exhaustion phenotype. In another rat model of liver transplantation (Ma et al., 2016), exosomes derived from immature DCs have been shown to amplify Tregs and prevent acute rejection.

## 4 pDC in the liver transplantation immune response

### 4.1 pDC in the liver transplant rejection

It is well known that pDCs can rapidly secrete large amounts of IFN-I in response to viral infection. In addition to secreting IFN-I, pDCs can express MHC-II molecules and costimulatory molecules CD40, CD80, and CD86, and can present antigens to CD4^+^ T cells, although not as effective as cDCs (Villadangos and Young, 2008; Reizis et al., 2011). Furthermore, pDCs can also regulate the survival of natural killer (NK) cells, DCs and macrophages through IFN-I, and expand CD4^+^ T cells and CD8^+^ T cells (Cervantes-Barragan et al., 2012; Brewitz et al., 2017). pDCs are considered to be a key factor in allograft rejection, but the underlying mechanism is still unclear. Ruben *et al.* demonstrated that pDC is an effective APC that can coordinate alloimmune responses and may play a key role in inducing chronic rejection in kidney transplantation (Ruben et al., 2018). The role of pDCs in solid organ rejection remain elusive. Experimental transplantation models have demonstrated that pDCs have the ability to present alloantigen (Björck et al., 2005; Ochando et al., 2006; Koyama et al., 2009). Another study showed that there was a large influx of pDCs in the renal tubulointerstitium at the time of rejection compared with the renal biopsy at the time of transplantation (Zuidwijk et al., 2012). In mice liver transplantation, if the donor is injected with FLT3L before transplantation to stimulate the generation of hepatic DCs (Drakes et al., 1997; Kingham et al., 2007), acute allograft rejection will occur in mice receiving liver transplantation without immunosuppression (Steptoe et al., 1997). This may be due to the fact that both cDCs (Steptoe et al., 1997) and pDCs (Kingham et al., 2007) mobilized by FLT3L increased the expression of CD80, CD86 and MHC-II molecules and enhanced the stimulatory ability of allogeneic T cells compared with normal hepatic DCs.

### 4.2 pDC in the liver transplant tolerance

Like cDCs, pDCs exhibit dual functions of immunogenicity and tolerance based on receptor connectivity and activation status. pDCs act as both innate antiviral immune effectors and inducers and regulators of adaptive immunity (Fonteneau et al., 2003), including hepatic T cell responses (Swiecki and Colonna, 2010). They drive the development of natural Tregs (Martín-Gayo et al., 2010), induce and maintain antigen-specific Tregs (Moseman et al., 2004; Palomares et al., 2012). Compared with lymphoid tissue pDCs, donor derived hepatic pDCs express high levels of DNAX-activating protein of 12 kDa (DAP12), triggering receptor expressed on myeloid cells 2 (TREM2) and a high proportion of T cell coinhibitory molecule PD-L1: costimulatory molecule CD80/86, which can attenuate graft infiltrating T effector cell responses, enhance forkhead box P3 (FOXP3)+Treg, and promote spontaneous tolerance of liver allograft in mice (Nakano et al., 2021). Compared with spleen-derived pDCs, mouse liver-derived pDCs exhibit an immature phenotype and lower levels of IL-12p70 secretion, and therefore, they exhibit reduced ability to present antigens or activate T cells (Kingham et al., 2007; Tokita et al., 2008). In addition, compared with spleen pDCs, liver pDCs secrete lower levels of IFN-I after cytosine phosphate guanosine (CpG) stimulation (Castellaneta et al., 2009), which can be explained by their relatively high expression of nucleotide-binding oligomerization domain-containing protein 2 (NOD2), a member of the nucleotide-binding oligomerization domain-like receptor (NLR) family. NOD2 can inhibit DC maturation and its ability to induce the proliferation of allogeneic T cells by inhibiting TLR signaling (Manicassamy and Pulendran, 2011). NOD2 can also interfere with TLR4 and TLR9 signaling pathways in pDCs through its ligands, but not in cDCs, resulting in decreased secretion of pro-inflammatory cytokines (IL-6, IL-12p70, TNF-α, and IFN-γ) (Castellaneta et al., 2009). What’s more, NOD2 ligation upregulated the coinhibitory PD-L1 expression on pDCs, resulting in its reduced ability to stimulate T cell proliferation (Castellaneta et al., 2009). pDCs promote tolerance *in vivo* by inducing energy deficiency or deletion of circulating T cells (Thomson and Knolle, 2010). Studies have shown that pDCs can induce the expression of IL-10 in Tregs, as well as release IFN-I (IFN-α/β) and IDO to attenuate the activation of allogeneic T cells (Cella et al., 1999; Munn et al., 2004; Ito et al., 2007). IDO has been shown to skew the development of naive CD4^+^ T cells toward the Treg lineage (Fallarino et al., 2006), which relies on cell-cell contact mediated mechanisms (Sharma et al., 2007). The expression of highly inducible co-stimulator ligand (ICOS-L) endows mature pDC with the ability to suppress effector T cells and increase IL-10-producing Treg cells (Campana et al., 2015).

## 5 Mo-DC in the liver transplantation immune response

Monocytes can be divided into two main types in mice and humans (Auffray et al., 2007; Yona et al., 2013; Guilliams et al., 2018): classical or inflammatory monocytes (Ly6C^hi^CX3CR1^int^ in mice and CD14^hi^CD16^-^ in human), nonclassical or patrolling monocytes (Ly6C^low^CX3CR1^hi^ in mice and CD14^low^CD16^+^ in human). At steady state, classical monocytes are stored in the bone marrow and other extramedullary sites, such as the spleen (Wolf et al., 2019). They can maintain the stability of tissue-resident macrophages in multiple organs, as well as the conversion to nonclassical monocytes (Guilliams et al., 2018). During infection and inflammation, classical or inflammatory monocytes can immediately deploy to infected or injured tissues to control infection, limit inflammatory damage and initiate tissue repair by producing macrophages or Mo-DCs (Boyette et al., 2017; Wolf et al., 2019). Nonclassical or patrolling monocytes are characterized by patrolling the circulation, clearing cellular debris and repairing the endothelium in homeostasis (Hanna et al., 2011; Carlin et al., 2013).

Most of our knowledge about Mo-DC comes from infection models. Under inflammatory conditions, classical monocyte-derived DC can become a large population that complements the range of steady-state DCs (Serbina et al., 2003; Le Borgne et al., 2006; León et al., 2007). Mo-DCs upregulate CD11c and MHC-II, but generally retain expression of monocyte markers such as Ly6C, Ly6B, CD64 (León et al., 2007; Rosas et al., 2010; Plantinga et al., 2013). They mediate effector functions through the production of T_h_1 cytokines TNF-α and IL-12, as well as direct cytotoxicity through NO production (Serbina et al., 2003; León et al., 2007; Goldszmid et al., 2012; Plantinga et al., 2013). In addition, Mo-DCs have phagocytic activity and the ability to process antigens, participating in the initiation of naive T cells or the reactivation of antigen experienced T cells (Mildner et al., 2013). Furthermore, it has been found in some tumor studies that the presence of Mo-DC is associated with the activation of CD8^+^ T cells and treatment success in some tumors (Rich et al., 2012; Kuhn et al., 2013; Kuhn et al., 2015). Mo-DC based vaccination also plays a role in various malignant tumors (Frankenberger et al., 2005; Javed et al., 2016; Van den Bergh et al., 2017).

Unlike classical monocytes, which play a major role in the site of inflammation (Serbina et al., 2003), nonclassical monocytes play a major role in sterile inflammation (for instance, in atherosclerotic plaques (Tacke et al., 2007)). In organ transplantation, the synergistic effect of allogeneic non-self and sterile inflammation may trigger the differentiation of nonclassical monocytes into DCs (Zhuang et al., 2016), nonclassical monocyte-derived DCs may also arise in the context of transplanted organs and may be involved in inducing allograft rejection (Chow et al., 2016). In a study of heart and kidney transplantation in mice, it was found that donor DCs would be rapidly replaced by recipient DCs after transplantation. Recipient nonclassical monocyte derived DCs played an important role in rejection by promoting the proliferation and survival of effector T cells in the graft (Zhuang et al., 2016). In another experimental study in mice, Mo-DCs was rapidly recruited after experimental heart and kidney transplantation, allogeneic grafts elicited persistent differentiation of monocytes to mature DCs that express IL-12, stimulate T cell proliferation and IFN-γ production, and precipitate graft rejection (Oberbarnscheidt et al., 2014). Similarly, *Zecher et al.* injected allogeneic splenocytes into the ear pinnae of recombination activating gene (RAG)^−/−^ mice, resulting in significantly greater skin swelling and infiltration by host bone marrow cells compared with injection of syngeneic splenocytes. This response is mediated by Gr-1 (Ly6C)^int^ monocytes, suggesting that they are able to discriminate between self and nonself tissue to initiate effector responses (Zecher et al., 2009). In another study, it was also observed that intravenous injection of allogeneic splenocytes in mice could stimulate the rapid (within 1 day) accumulation of splenic Mo-DCs more than syngeneic splenocytes or nothing injection. These findings suggest that (Chow et al., 2016) Mo-DCs have a potentially important role in responding to allogeneic stimuli, such as those occurring in the context of organ transplantation. In addition, in mouse pancreas transplantation, Mo-DCs can impair the early graft function after islet allograft transplantation, and the graft function of recipients with Mo-DC removed is improved as early as the first day after transplantation, which strongly suggests that Mo-DCs can participate in the early injury of islet allografts (Chow et al., 2017). In an experimental study of bone marrow transplantation (Asiedu et al., 2002), non-human primate (NHP) mature Mo-DCs can be genetically engineered as an alloantigen specific cellular immunosuppressant, which has the potential to promote the induction of allograft tolerance *in vivo*. What’s more, In a recent study (Lee et al., 2021), due to the relatively simple culture process and more efficient Treg expansion capacity of stimulated mature Mo-DCs, they may be a viable alternative for producing alloreactive Tregs for clinical use. But until recently, experimental research on Mo-DC in liver transplantation was extremely rare. In a study of human liver transplantation (Shariat et al., 2014), researchers found that compared with healthy individuals, the gene expression of IL-12 and TLR2 secreted by Mo-DCs in liver transplant patients were significantly increased. Signaling through TLR2 can induce DCs to mature in a myeloid differentiation primary response 88 (MyD88) dependent or independent manner, ultimately leading to the release of proinflammatory cytokines (Chinen and Buckley, 2010). In addition, the increase of TLR2 and TLR4 levels can also be used for the early prediction of acute rejection after liver transplantation (Deng et al., 2007). IL-12 can induce the development and inflammatory process of T_h_1 cells in transplanted patients (Goriely and Goldman, 2008), which may contribute to the occurrence of liver transplantation rejection. Overall, although the role of Mo-DC in infection and inflammation has been increasingly understood, their special role in organ transplantation, especially in the case of liver transplantation, still needs more research to explore.

## 6 The latest progress of DC based treatment

The use of donor or host-derived tolerogenic dendritic cell (Tol-DC) or *in situ* targeted DC for cell therapy to promote its tolerance is an emerging method to reduce the use of systemic IS in transplant patients and promote donor-specific tolerance (Ochando and Braza, 2017; Marín et al., 2018). Tol-DC refers to some DCs that can inhibit immune responses, including immature DC (imDC), regulatory DC (DCreg), maturation resistant or alternatively activated DC (Morelli and Thomson, 2007). Tol-DCs are characterized by low expression of MHC-II and co-stimulatory molecules CD80/CD86 and CD40, high expression of anti-inflammatory cytokines such as IL-10 and transforming growth factor beta (TGF-β), and low expression of pro-inflammatory cytokines such as IL-12p70 (Maldonado and von Andrian, 2010; Marín et al., 2018). In addition, Tol-DCs also have low antigen-presenting function and participate in immune tolerance by inducing reactive T cell weakness and apoptosis (Choo et al., 2017; Spiering et al., 2019). Tol-DC has been extensively studied in preclinical models and is very effective in limiting autoimmune diseases (Hilkens et al., 2010) or allograft rejection in transplantation (Ochando et al., 2020; Chen et al., 2023).

**FIGURE 2:**
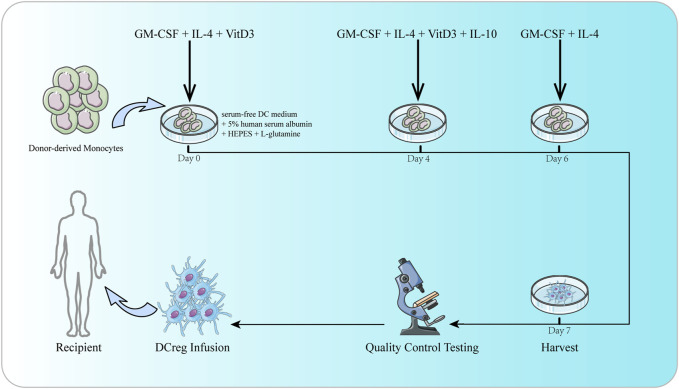
The culture of DCreg. Donor-derived monocytes are resuspended in DC culture media consisting of serum-free DC medium, 5% human serum albumin, HEPES, and L-glutamine. GM-CSF, IL-4 and VitD3 are added on days 0 and 4. IL-10 is also added on day 4. On day 6, culture media is replenished with DC media supplemented with GM-CSF and IL-4. DCreg products are harvested on day 7. Before infusion, DCreg products are subjected to rigorous quality control testing to ensure purity, sterility, yield and viability.

Extensive studies in rodent and NHP models have shown that adoptively transferred regulatory immune cells can promote transplant tolerance. DCreg is an important candidate for adoptive cell therapy in organ transplantation (Moreau et al., 2017; Thomson et al., 2019), which can suppress T cell responses by inducing T cell weakness or apoptosis (Lu et al., 1997; Lu and Thomson, 2002; Zahorchak et al., 2018). What’s more, DCregs also have the potential to retain, expand or induce Treg (Morelli and Thomson, 2007; Huang et al., 2010; Raker et al., 2015). DCregs were generated in granulocyte-macrophage colony-stimulating factor (GM-CSF) and IL-4 from rodent and human bone marrow precursor cells or human circulating blood monocytes *in vitro* (Zahorchak et al., 2023). In the culture of DCreg, one or more agents, cytokines or growth factors or genetic engineering (Bonham et al., 2002) methods are added to inhibit DC maturation and enhance its tolerance (Hackstein and Thomson, 2004). These agents include immunomodulatory drugs [such as vitamin D3 (VitD3), corticosteroids, rapamycin, cyclosporine, tacrolimus, and aspirin], cytokines or growth factors [such as hepatocyte growth factor (HGF), vasoactive intestinal peptide (VIP), thymic stromal lymphopoietin, PGE2, IL-10, and TGF- β] (Raker et al., 2015; Thomson et al., 2019). However, there is still no consensus on the best protocol for generating clinical-grade human DCreg (Navarro-Barriuso et al., 2018; Thomson et al., 2019). DCreg infusion improves the incidence of safe withdrawal of IS and operational tolerance in human liver transplantation (Thomson et al., 2019). The safety, feasibility and potential efficacy of autologous DCreg infusion has been confirmed in the treatment of various diseases, including type-1 diabetes, rheumatoid arthritis, Crohn’s colitis, multiple sclerosis (Giannoukakis et al., 2011; Jauregui-Amezaga et al., 2015; Bell et al., 2017; Zubizarreta et al., 2019). Studies have described the rationale for the first human trial of donor-derived DCreg injected before living donor liver transplantation (LDLT) in combination with triple IS (steroid hormones, mycophenolate mofetil and tacrolimus) to promote operational tolerance (Zahorchak et al., 2018). In another study (Macedo et al., 2021), infusion of donor monocyte-derived DCregs in the first 7 days of LDLT can induce changes in host APC, memory CD8+T cell and Treg, which may be beneficial to regulate immune reactivity during transplantation, thereby promoting operational tolerance. In the latest study of manufacturing GMP grade DCreg for 27 liver transplant recipients (Zahorchak et al., 2023), under GMP conditions, circulating monocytes can easily produce high-purity DCreg that meets a series of quality standards to attain the target cell number injected into potential organ transplant recipients (Figure 2). Two clinical trials of DCreg in liver transplantation (NCT03164265 and NCT04208919) are ongoing to evaluate the safety and efficacy of donor derived DCreg in LDLT through a single infusion. The registered clinical trials targeting DC in transplantation are shown in Table 2.

**TABLE 2:** Registered clinical trials targeting DC in liver or kidney transplantation.

Cell type	Conditions	Intervention/treatment	Trial id	Status
Autologous tolerogenic dendritic cell	living donor renal transplantation	Autologous tol-DC treatment occurs the day before transplantation into recipients also recipients of a living donor renal transplantation	NCT02252055	Completed
Donor blood monocyte-derived regulatory dendritic cell	living donor renal transplantation	single infusion of donor-derived DCreg 1 week before living donor renal transplantation	NCT03726307	Recruiting
Donor blood monocyte-derived regulatory dendritic cell	living donor liver transplantation	single infusion of donor-derived DCreg 1 week prior to the initiation of immunosuppression weaning	NCT04208919	Active, not recruiting
Donor blood monocyte-derived regulatory dendritic cell	living donor liver transplantation	single infusion of donor-derived DCreg 1 week before living donor liver transplantation	NCT03164265	Active, not recruiting

What’s more, a recent study have used the optimization of physical and chemical properties of nanoparticles to design DC targeted nanomedicines for immune tolerance in organ transplantation and autoimmune diseases (Cifuentes-Rius et al., 2021). Targeting specific receptors on DC using nanomedicines not only helps to enhance absorption, but also has the ability of antigen cross presentation, thereby promoting peripheral tolerance. Despite its great potential, this method is still in its infancy, and its persistence, safety, and complexity related to the immune system and multiple mechanisms may limit the development of this method.

## 7 Conclusion and future prospects

In this article, we described the subgroups, development, phenotype, migration and function of DC, and the roles of these DC subsets, including cDC, pDC, and Mo-DC in liver transplant rejection and tolerance. During rejection, cDC plays an important role in the initiation of immune rejection by presenting donor antigens. pDC, as a weaker APC, cannot be overlooked as they also enhance the stimulation ability of allogeneic T cells by expressing MHC-II molecules and costimulatory molecules. Now, increasingly evidence suggested the Mo-DC is the major mediator to activate alloreactive T cell in the graft, but their role in liver transplantation is not fully revealed. In the future, more effort is needed to explore the role of Mo-DC in alloresponse in the liver allograft.

By adopting an immature phenotype, both cDC and pDC can promote T cell apoptosis and generate Tregs to promote immune tolerance. Adoptive transfer of the DCreg or Tol-DC exhibiting immature phenotype induces tolerance, but the best clinical grade DCreg or Tol-DC production scheme is still needed.

In conclusion, these discussions provide useful resources for better understanding the biology of DC and improving the immune adaptability of transplant patients by manipulating DC.
